# Numerical entropy analysis of MHD electro-osmotic flow of peristaltic movement in a nanofluid

**DOI:** 10.1016/j.heliyon.2024.e27185

**Published:** 2024-03-01

**Authors:** M. Gnaneswara Reddy, K. Venugopal Reddy, Basma Souayeh, H. Fayaz

**Affiliations:** a Department of Mathematics, Acharya Nagarjuna University Campus, Ongole, 523 001, AP, India; b Department of Mathematics, Anurag University, Hyderabad, 500100, India; c Department of Physics, College of Science, King Faisal University, PO Box 400, Al-Ahsa, 31982, Saudi Arabia; d Department of Physics, Laboratory of Fluid Mechanics, Faculty of Sciences of Tunis, University of Tunis El Manar, 2092, Tunis, Tunisia; e Modeling Evolutionary Algorithms Simulation and Artificial Intelligence, Faculty of Electrical and Electronics Engineering, Ton Duc Thang University, Ho Chi Minh City, Vietnam

**Keywords:** Peristalsis, Electro-osmosis flow, MHD, Entropy generation, Nanofluid

## Abstract

The present study investigates the MHD electro-osmotic flow of entropy generation analysis for peristaltic movement in a nanofluid with temperature-dependent viscosity. Long wavelengths, i.e., The magnitude of a wave's energy corresponds directly to its frequency while being inversely related to its wavelength in terms of velocity, temperature, and concentration, govern and confine the flow stream in the laminar region. Ohmic heating and hall effects are also included. Graphs are used to obtain and examine numerical solutions for axial velocity, temperature, concentration, Bejan number, and entropy generation. The effects of this research can help to improve pumping and gastrointestinal movements in different engineering devices. Debye–Huckel and lubrication approximations are studied to access the Boltzmann distribution of electric potential across an electric double layer. The investigations of an existing model are important in illuminating the microfluidics machinery used at the micro level for various transport phenomena in which fluids as well as particles are transported together. The current study has many applications and can be further extended to a three-dimensional profile with appropriate modifications and assumptions. When studying entropy generation, it is essential to examine the irreversible factors, while also taking into account the velocity and thermal slip conditions at channel boundaries. Moreover, the concept of entropy generation holds significant importance in comprehending various biological phenomena. Hence, the current research holds promising implications for both industrial and medical fields. The entropy generation is minimum at left wall of the channel for negative values of Helmholtz-Smoluchowski velocity.

## 1 Introduction

Electro-osmosis has become extremely important to researchers in recent years. Electro-osmosis is the phenomenon where the movement of liquids is aided by an external electric field across a power-law fluids [1]. During the 1980s, the MEMS were developed in the initial times. These devices have been widely used in various industries, particularly in the field of medicine and biology. One specific type of MEMS device, known as lab-on-chip (LOC), has emerged as a promising microfluidic technology for applications such as clinical analysis and detective biological contagion. The present microfluidics are very useful which are fast, efficient, and consistent performance may have led to a significant shift towards small, easy to use, and affordable devices in DNA assays and drug distribution. Electro-osmosis occurs naturally in many biological systems and medical. It also occurs in botanical systems [2], electroosmotic flow in non-Darcy porous medium [3] (interstitial fluid rich in ions), estimates of flow in bio-fluidic devices [4], micro-channel fluids of couple stresses [5], and physiological vessels [6]. This effect has been utilized in fractional Jeffrey fluid industrial separation processes. [7], electrokinetically modulated [8], Williamson fluid in an asymmetric channel [9], titanium magneto-nanoparticles [10] and in particular porous micro vessels altered by electro hydrodynamic [11]. Microfluidic systems with electro-osmosis often pour fluid throughout the entire system, subject to electro-osmosis. The motion of electrolytes from the channel along a charged boundary is defined by the applied voltage. Electro-osmosis is used in many medical, industrial and biological processes, such as channel flow, skin human movement, porous membranes, dialysis of fluid, botanical procedures and separation methods. Microelectronics and Microfluidics have also led to many different applications, including biological as well as microelectronic refrigeration.

The rapid advancement of nanotechnology has brought scientific research up to date and created an important area of concern for researchers. The term “nanofluids" refers to an increase in thermal conductivity from 1 nm to 100 nm. Solid particles and their oxides, nitrides, graphene, carbides, and carbon nanotubes are all examples of nanoparticles. Nanoparticles dispersion (including metal oxides (Titania), non-metallic carbon nanotubes or carbides (Silicon), silver and copper) with high heat capacities and thermal conductivity enhance conventional fluids' thermal performance. Nanofluids have a remarkable ability to improve the energy efficiency and heat transfer rate of thermal systems. As a result, they are applicable to device compactness. Dharmendra and Anwar [12] deliberated the application in drug delivery systems. Prakash and Ramesh [13] examined the peristaltic transport of sutterby nanofluids. An additional significant research endeavor focused on the dispersion of various nanoparticles into water-based nanofluids. The study observed an improvement in the thermal effectiveness of the nanofluid through the incorporation of nanoparticles into the standard liquid. The research yielded valuable insights into nanofluids and their potential applications can be seen in Refs. [[14], [15], [16]]. MHD effects on hyperbolic tangent nanofluid peristalsis movement in an inclined channel under joule heating and slip circumstances by Hayat et al. [17]. Prakash and Kothandapani [18] described the effects of magnetic field and thermal radiation parameter on Williamson nanofluid peristaltic motion in a tapered asymmetric channel. Mallick et al. [19] studied the influence of joule heating and hall current on entropy generation during thermo radiative transport of nanofluids in a porous microchannel generated by electrokinetic processes. Dey and Shit [20] investigated the transport of fluid with specific properties, subject to both interfacial slip and electro-osmotic effects along with exchange of heat, occurs in microchannels under the influence of magnetic fields. Nanofluids, known for their superior heat transfer properties, are applied in various heat exchanger systems like electronic cooling setups, chips, and radiators. High-tending nanofluids have applications in biomedicine, such as cryosurgery, in vitro cell therapy, cancer diagnosis, tumour cell destruction, neuro-electronic interfaces, and so on. Considering to the significance of these applications in nature, researchers have focused their attention on hybrid nanofluids.

Unsteady peristaltic motion is part of one of the most important phenomena because of its numerous applications in biomedicine, the chemical industry, engineering, and physiology. Electro-osmotic transport phenomena are commonly observed in the human body, such as ovum movement, the vasomotion of tiny blood vessels, the fallopian tube, and so on. Peristalsis is used by finger pumps and roller pumping systems. The earliest activities in this direction were made by Latham [21] and Shapiro et al. [22]. Subsequent experiments on peristalsis have been documented in studies [[23], [24], [25], [26], [27], [28], [29], [30]]. It is worth mentioning that the magnetohydrodynamic peristaltic flow of fluids finds extensive applications in engineering and various industries. The magnetic field is utilized for treating inflammations, cancerous tumors, hyperthermia, ulcerations, blood loss during surgeries, and numerous intestinal and ureteral diseases. Since these real-world uses are so significant, researchers have focused their efforts on using magnetic fields in biomedical science and engineering. Several of these applications include the use of MRIs, NMRIs, and MRTs (magnetic resonance tomography) to visualize physiological processes within the human body. Sensitive sensors created from GMR (giant magneto resistive) technology, work on hybrid nanofluids, polymer technology, aerodynamic heating processes, MHD generators, MHD accelerators, and pumps and play a significant role in detecting and facilitating peristaltic movements in the intestines, fallopian tubes, vas deferens, and other areas, showcasing the substantial industrial and medical implications of MHD. Gnaneswara Reddy et al. [31,32] considered the hydromagnetic peristaltic motion of a non-Newtonian fluid in a various asymmetric channel. Makinde et al. [33] made a study about the impact of effects of thermal radiation on MHD peristaltic motion of Walters-B Fluid with warmness supply and Slip conditions. Pandey and Chaube [34] include analysis regarding peristaltic shipping in the presence of an outside magnetic area of a micropolar fluid via a porous medium.

Most of the methods to enact the estimation model for the air-earth tunnel consist mainly of heat exchangers, analytical, and numerical methods. Where the significant computational efficiency can epitomize and summarize the definite expressions between the main parameters However, the boundary conditions follow strict harmonic laws for the most part. This leads to large errors that are inconsistent with the actual situation. Representative works in this regard are Abbasi et al. [35] who also considered the MHD peristaltic movement of nanofluid of entropy generation analysis through a non-uniform asymmetric channel with variable thermal conductivity. Abbasi et al. [36] investigated the analysis of peristaltic nanofluid flow with ohmic heating and hall current with entropy generation. Das and Das [37] who reported the elements in the turbulent ionic tribological liquid flow in the squeezed/split channel provide the basis for a high-energy magnetic field. Das et al. [38] examined the effect of ternary hybrid nanofluid EDL on mixed magneto-convection in a vertical channel. Das and Barman [39] discussed the peristaltic microchannel electro-osmosis of an ionic hybrid nanofluid with corridorans ion-slip currents. They have stated that when the magnetic field gets stronger, the fluid's cost decreases along with the drift. They have stated that the fluid float's velocity decreases as the magnetic subject grows. Most recently [[40], [41], [42], [43], [44], [45], [46]], investigated the blood clotting consequences on peristaltic viscoelastic nano-fluids via annulus in MHD. They stated that the drift speed decreases with the peak of blood clot.

Any thermal device in trendy is connected to the production of irreversible, which determines how efficiently its thermal properties work. The dynamics of thermal structures are determined by a multitude of elements, some of which are irreversible. The work aims to elucidate new uses in the realm of microfluidic pumping procedures, with the expectation of inspiring fellow researchers to delve into the captivating field of biomedical engineering. The non-Newtonian nanofluid (Carreau-Yasuda) moving in a tapered channel as a result of blood's electrical forces accompanied by entropy generation was communicated by Abbasi [47]. Some latest packages of entropy era may be determined in Refs. [[48], [49], [50], [51]].

Electro-osmosis induced transport has emerged as a novel method for designing micro-fluidic systems with significant implications in the fields of mechanics and biomechanics. The current paper discusses a mathematical model for Magneto hydrodynamics (MHD) entropy generation analysis with thermal conductivity in the electro-osmotic flow (EOF) of nanofluid peristalsis movement through a non-uniform, asymmetric channel with various zeta potentials. The governing equations are simplified by employing dependable approximations, specifically the Debye-Huckel and lubrication approximations. The resulting equations are then resolved by employing the bvp4c function included with the MATLAB 2012b software. Graphs depict the impact of relevant parameters on flow: axial velocity distribution, temperature distribution, concentration distribution, Bejan number, and entropy generation over the channel. This research is expected to shed light on novel biological microfluidics applications. Additionally, it has been noted that in the region of the channel walls, there are minor, irreversible consequences for both increased variable viscosity and thermal conductivity. The aforementioned model is anticipated to be advantageous for numerous biomicrofluidics applications.

## 2 Modelling

### 2.1 Electrical model

The familiar Poisson equation of the electro potential with in the microchannel is given by:

(1) $$\nabla^{2}\bar{E}=-\frac{{\bar{\rho }}_{e}}{\varepsilon }$$

where ${\bar{\rho }}_{e}=\left({\bar{n}}^{+}-{\bar{n}}^{-}\right)ez$ is the density of the electrical, $n_{0}$ and a valence of $z_{+}$ and $z_{-}$, e represents elementary charge, ${\bar{n}}_{-}$ and ${\bar{n}}_{+}$ are containing number density having negative and positive ions (concentration of bulk) respectively.

The Poisson Eq. (1) now assumes the form:

(2) $$\frac{\partial^{2}\bar{E}}{\partial y^{2}}=-\kappa^{2}\left(\frac{{\bar{n}}_{+}-{\bar{n}}_{-}}{2}\right)$$

where $\lambda_{d}=\frac{d}{\kappa }$ is the electrical double layer's usual thickness (EDL) and $\kappa =dez\sqrt{\frac{2n_{0}}{\varepsilon K_{B}T_{e}}}$ is the electroosmotic parameter

Assume that the Nernst-Planck equation is reduced to the following form:

(3) $${\bar{n}}_{\pm }=e^{\mp \bar{E}}$$

The modified equation of (2) can now be written as $\frac{\partial^{2}\bar{E}}{\partial y^{2}}=-\kappa^{2}\;\sinh\bar{E}$. Debye-Hückel linearization is employed to reduce the Poisson-Boltzmann equation to:

(4) $$\frac{\partial^{2}\bar{E}}{\partial y^{2}}=\kappa^{2}\bar{E}$$

### 2.2 Nanofluid model

**Fig. 1:**
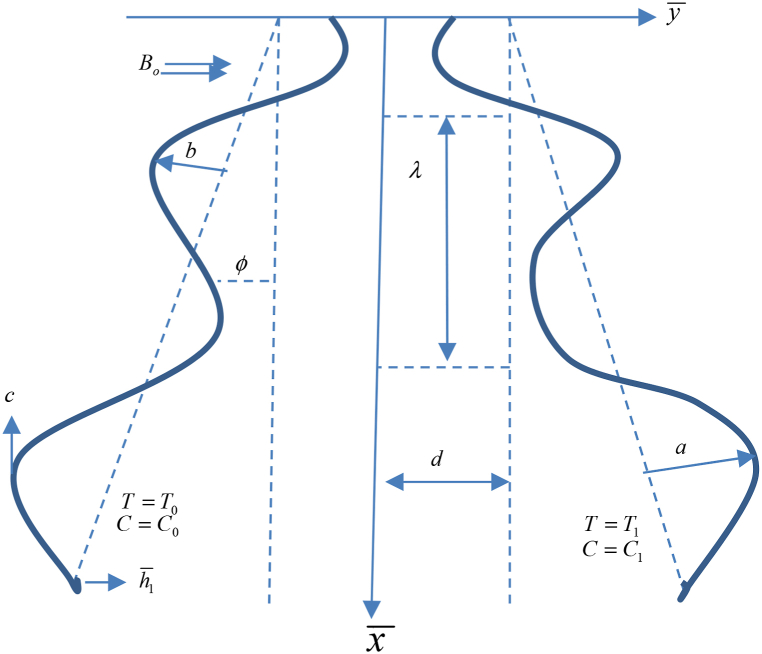
Flow geometry.

An Electro Osmotic flow of a fluid of viscosity by a peristaltic wave's movement of nanofluids in an asymmetric non-uniform channel of width 2d is considered. In this case, the coordinate system is chosen with the condition that one axis be selected along the length of the channel and the other be taken normal to it. Because of the magnetized low Reynolds number, the induced magnetic and electric fields are taken for assumed. Flow analysis is displayed in Fig. 1.

The waves of the mathematical form are expressed as

(5) $${\bar{h}}_{1}\left(\bar{t}+\bar{x}\right)=m\bar{x}+d+a_{1}\;\sin\left(\frac{2\pi }{\lambda }\left(-c\bar{t}+\bar{x}\right)\right)$$

(6) $${\bar{h}}_{2}\left(\bar{t}+\bar{x}\right)=-\bar{m}\bar{x}-d-b_{1}\;\sin\left(\in +\frac{2\pi }{\lambda }\left(-c\bar{t}+\bar{x}\right)\right)$$

Here ${\bar{h}}_{1},{\bar{h}}_{2},a_{1},b_{1},\bar{t}$ and λ represents left wall, right wall, left and right amplitude of the asymmetric channel, time and wave length respectively.

(7) $$a_{1}^{2}+b_{1}^{2}+2a_{1}b_{1}\;\cos\left(\varepsilon \right)\leq {\left(2d\right)}^{2}$$

The existing flow pattern's governing equations are provided as [27,35] and

(8) $$\frac{\partial \bar{u}}{\partial x^{*}}+\frac{\partial \bar{u}}{\partial y^{*}}=0$$

(9) $$\rho_{f}\left(\frac{\partial }{\partial t}+\bar{u}\frac{\partial }{\partial x^{*}}+\bar{v}\frac{\partial }{\partial y^{*}}\right)\bar{u}=-\frac{\partial \bar{P}}{\partial x^{*}}+\left(\frac{\partial {\bar{S}}_{x^{*}x^{*}}}{\partial x*}+\frac{\partial {\bar{S}}_{x^{*}y^{*}}}{\partial y*}\right)+\left(1-C_{o}\right)\left(T-T_{m}\right)\rho_{f}g\alpha^{*}+\left(\rho_{p}-\rho_{f}\right)\left(C-C_{m}\right)g\omega^{*}-\bar{u}B_{o}^{2}\sigma_{f}+{\bar{\rho }}_{e}E_{x}$$

(10) $$\rho_{f}\left(\frac{\partial }{\partial t}+\bar{u}\frac{\partial }{\partial x^{*}}+\bar{v}\frac{\partial }{\partial y^{*}}\right)\bar{v}=-\frac{\partial \bar{P}}{\partial y^{*}}+\left(\frac{\partial {\bar{S}}_{y^{*}y^{*}}}{\partial y^{*}}+\frac{\partial {\bar{S}}_{y^{*}x^{*}}}{\partial x^{*}}\right)$$

(11) $${\left(\rho c^{\prime }\right)}_{f}\left(\frac{\partial }{\partial t}+\bar{u}\frac{\partial }{\partial x^{*}}+\bar{v}\frac{\partial }{\partial y^{*}}\right)T=\bar{\nabla }.\left[\left(\bar{\nabla }T\right)\bar{K}\left(T\right)\right]+\bar{S}.L+\sigma_{f}U^{2}B_{O}^{2}+\tau {\left(\rho c^{\prime }\right)}_{f}D_{B}\left(\frac{\partial C}{\partial x^{*}}\frac{\partial T}{\partial x^{*}}+\frac{\partial C}{\partial y^{*}}\frac{\partial T}{\partial y^{*}}\right)+\frac{{\left(\rho c^{\prime }\right)}_{f}\tau D_{T}}{T_{m}}\left({\left(\frac{\partial T}{\partial y^{*}}\right)}^{2}+{\left(\frac{\partial T}{\partial x^{*}}\right)}^{2}\right)$$

(12) $$\left(\frac{\partial }{\partial t}+u^{*}\frac{\partial }{\partial x^{*}}+v^{*}\frac{\partial }{\partial y^{*}}\right)C=D_{B}\left(\frac{\partial^{2}C}{\partial {y^{*}}^{2}}+\frac{\partial^{2}C}{\partial {x^{*}}^{2}}\right)+\frac{D_{T}}{T_{m}}\left(\frac{\partial^{2}T}{\partial {y^{*}}^{2}}+\frac{\partial^{2}T}{\partial {x^{*}}^{2}}\right)$$

In which the stress tensor ${\bar{S}}_{ij}$ components exhibit the non-Newtonian behaviour of nanofluids as represented by the above equations, $\rho_{f}$ refers to the fluid density, g refers to the gravitational acceleration, $\bar{K}\left(T\right)$ refers to the fluid's thermal conductivity, $\bar{P}$ refers to the pressure of the fluid, $C_{f}$ refers to the specific heat of liquid, T refers to the fluid temperature, C refers to the concentration, $T_{m}\left(=\frac{T_{o}+T_{1}}{2}\right)$ refers to the channel walls mean temperature, $C_{m}\left(=\frac{C_{1}+C_{0}}{2}\right)$ refers to the mean concentration of the channel walls. $\bar{S}.L$ refers to the viscous heating, L denotes the gradient of velocity, $a^{*}$ and $\omega^{*}$ represents the thermal and concentration expansion coefficients, $D_{T}$ and $D_{B}$ refers to the thermal diffusivity and mass diffusivity, $\tau \left(=\frac{{\left(\rho C\right)}_{p}}{{\left(\rho C\right)}_{f}}\right)$ designates “the ratio of effective heat capacity of the nanoparticles to the base fluid”.

Here, $C_{o}$, $T_{o}$ and $C_{1}$, $T_{1}$ are the concentration and temperature at left and right walls.

The extra stress tensor of nanofluid is given by

(13) $$\bar{S}=\mu \left(\overset{⌢}{\gamma }\right)A_{1}\text{.}$$

Where $\mu \left(\overset{⌢}{\gamma }\right)$ the apparent viscosity and $A_{1}$ indicates the “first Rivlin – Erickson tensor” and is given as:

(14) $$\overset{⌢}{\gamma }=\sqrt{2trD^{2}},D=\frac{1}{2}A_{1}\;\text{and}\;A_{1}=grad\;V+{\left(grad\;V\right)}^{T}$$

(15) $$\mu \left(\overset{⌢}{\gamma }\right)=\mu_{\infty }+\left(\mu_{o}-\mu_{\infty }\right){\left[1+{\left(\alpha \gamma \right)}^{a}\right]}^{\frac{n-1}{n}}$$

Here, $\mu_{o}$ and $\mu_{\infty }$ denote the zero and infinite shear rates of viscosity α, gradV , n, $\overset{⌢}{\gamma }$ represents velocity gradient fluid variable, non-dimensional power law index shear rate.

Define the following dimensionless parameters and flow variables: the

(16) $$\bar{u}=\frac{u^{*}}{c},\bar{v}=\frac{v^{*}}{c},t=\frac{ct^{\prime }}{\lambda },y^{*}=\frac{\bar{y}}{d},\delta =\frac{d}{\lambda },h_{1}=\frac{{\bar{h}}_{1}}{d},h_{2}=\frac{{\bar{h}}_{2}}{d},a=\frac{a_{1}}{d},b=\frac{b_{1}}{d},p=\frac{d^{2}\bar{p}}{c\lambda \mu_{o}},\xi =\xi_{o}T_{o}$$

$$S_{xy}=\frac{\bar{d}\;{\bar{S}}_{\bar{x}\bar{y}}}{\mu_{o}c},K\left(\theta \right)=\frac{\bar{K}\left(T\right)}{K_{O}},M^{2}=\frac{\sigma_{f}}{\mu_{o}}B_{o}^{2}d^{2},\beta =\frac{\mu_{\infty }}{\mu_{o}},\text{Re}=\frac{\rho_{f}cd}{\mu_{o}},\mathrm{Pr}=\frac{\mu_{o}C_{f}^{\prime }}{K_{o}},Ec=\frac{c^{2}}{C_{f}\left(T_{1}-T_{0}\right)},x^{*}=\frac{\bar{x}}{\lambda }$$

$$\varphi =\frac{C-C_{m}}{C_{1}-C_{0}},Br=\mathrm{Pr}\;Ec,N_{t}=\frac{\tau D_{T}\left(T_{1}-T_{0}\right)}{\nu T_{m}},N_{b}=\frac{\tau D_{B}\left(C_{1}-C_{0}\right)}{\nu },G_{t}=\frac{d^{2}\rho_{f}ga^{*}\left(1-C_{o}\right)\left(T_{1}-T_{0}\right)}{\mu_{o}c}\text{,}$$

$$G_{c}=\frac{d^{2}\left(\rho_{p}-\rho_{f}\right)g\omega^{*}\left(C_{1}-C_{0}\right)}{\mu_{o}c},We=\frac{ac}{d},Sc=\frac{\upsilon }{D_{B}},u=\psi_{y},v=-\delta \psi_{x},\theta =\frac{T-T_{m}}{T_{1}-T_{o}}$$

The nanofluid temperature of the thermal conductance is given by

(17) $$\bar{K}\left(T\right)=K_{o}\left(1+\xi_{o}\left(T-T_{m}\right)\right)$$

Where, $\xi_{o}$ depicts the fixed temperature of the dimensional thermal conductance.

By employing Eqs. (16), (17) in Eqs. (8)–(12), we have

(18) $$\text{Re}\left[\delta u\frac{\partial u}{\partial x}+v\frac{\partial u}{\partial y}+\delta \frac{\partial u}{\partial t}\right]=-\frac{\partial p}{\partial x}+\delta \frac{\partial }{\partial x}\left(S_{xx}\right)+\frac{\partial }{\partial y}\left(S_{xy}\right)-M^{2}u+G_{t}\theta +G_{c}\varphi +U_{HS}\;\frac{\partial^{2}E}{\partial y^{2}}$$

(19) $$\text{Re}\delta \left[\delta u\frac{\partial v}{\partial x}+v\frac{\partial v}{\partial y}+\delta \frac{\partial v}{\partial t}\right]=-\frac{\partial p}{\partial y}+\delta^{2}\frac{\partial }{\partial x}\left(S_{yx}\right)+\delta \frac{\partial }{\partial y}\left(S_{yy}\right)$$

(20) $$\text{Re}\left[\delta u\frac{\partial \theta }{\partial x}+v\frac{\partial \theta }{\partial y}+\delta \frac{\partial \theta }{\partial t}\right]=\frac{1}{\mathrm{Pr}}\delta \frac{\partial }{\partial x}\left[\delta \left(1+\xi \theta \right)\frac{\partial \theta }{\partial x}+\left(1+\xi \theta \right)\frac{\partial \theta }{\partial y}\right]+\frac{1}{\mathrm{Pr}}\frac{\partial }{\partial y}\left[\delta \left(1+\xi \theta \right)\frac{\partial \theta }{\partial x}+\left(1+\xi \theta \right)\frac{\partial \theta }{\partial y}\right]+N_{b}\left[\delta^{2}\frac{\partial \varphi }{\partial x}\frac{\partial \theta }{\partial x}+\frac{\partial \varphi }{\partial y}\frac{\partial \theta }{\partial y}\right]+N_{t}\left[\delta^{2}{\left(\frac{\partial \theta }{\partial x}\right)}^{2}+{\left(\frac{\partial \theta }{\partial y}\right)}^{2}\right]+E_{c}\left[\frac{\partial u}{\partial y}S_{xy}+\delta \frac{\partial v}{\partial x}S_{yx}\right]+EcM^{2}u^{2}$$

(21) $$\text{Re}Sc\left[\delta \frac{\partial \varphi }{\partial t}+\delta u\frac{\partial \varphi }{\partial x}+\nu \frac{\partial \varphi }{\partial y}\right]=\delta^{2}\frac{\partial^{2}\varphi }{\partial x^{2}}+\frac{\partial^{2}\varphi }{\partial y^{2}}+\frac{N_{t}}{N_{b}}\left[\delta^{2}\frac{\partial^{2}\theta }{\partial x^{2}}+\frac{\partial^{2}\theta }{\partial y^{2}}\right]$$

Where $G_{t}$ , Pr, $G_{c}$, Ec, M, Br, We are thermal Grashof number, Prandtl, concentration Grashof number, Eckert, Hartman, Brikman and Weissenberg number.

By utilizing the larger wavelength theory and smaller Reynolds number, Eqs. (18)–(21) take the form:

(22) $$\frac{\partial p}{\partial x}=\frac{\partial S_{xy}}{\partial y}-M^{2}\frac{\partial \psi }{\partial y}+G_{t}\theta +G_{c}\varphi +U_{HS}\;\frac{\partial^{2}E}{\partial y^{2}}$$

(23) $$\frac{\partial p}{\partial y}=0$$

(24) $$\frac{\partial }{\partial y}\left[\left(1+\xi \theta \right)\frac{\partial \theta }{\partial y}\right]+BrM^{2}{\left(\frac{\partial \psi }{\partial y}\right)}^{2}+N_{b}\;\mathrm{Pr}\left(\frac{\partial \varphi }{\partial y}\frac{\partial \theta }{\partial y}\right)+N_{t}\;\mathrm{Pr}\;{\left(\frac{\partial \theta }{\partial y}\right)}^{2}+Br\varphi =0$$

(25) $$\frac{\partial^{2}\varphi }{\partial y^{2}}+\frac{N_{t}}{N_{b}}\frac{\partial^{2}\theta }{\partial y^{2}}=0$$

(26) $$\frac{\partial^{2}E}{\partial y^{2}}-\kappa^{2}E=0\text{.}$$

where

(27) $$\varphi ={\left(\frac{\partial^{2}\psi }{\partial y^{2}}\right)}^{2}\left(1+\left(1-\beta \right)\left(n-1\right)\frac{\partial^{2}\psi }{\partial y^{2}}We\right)$$

The stress tensor in dimensionless form is given by Ref. [35]:

(28) $$\psi =\frac{F}{2},\frac{\partial \psi }{\partial y}=0,\theta =-\frac{1}{2}\;\text{and}\;\varphi =-\frac{1}{2}\;\text{at}\;y=mx+1+a\;\sin\left[2\pi \left(-t+x\right)\right]=h_{1}$$

(29) $$\psi =-\frac{F}{2},\frac{\partial \psi }{\partial y}=0,\theta =\frac{1}{2}\;\text{and}\;\varphi =\frac{1}{2}\;\text{at}\;y=-mx-1-b\;\sin\left[\varepsilon +2\pi \left(-t+x\right)\right]=h_{2}$$

The stress tensor in dimensionless form is given by:

(30) $$S_{xy}=\frac{\partial^{2}\psi }{\partial y^{2}}\left[1+\left(1-\beta \right)\left(n-1\right)We\frac{\partial^{2}\psi }{\partial y^{2}}\right]$$

Also, the flow rate which is in dimensionless form as

(31) F(t,x)=asin[ε+2π(−t+x)]+bsin[2π(−t+x)]+η

where,

(32) $$F=\int_{h_{2}}^{h_{1}}\frac{\partial \psi }{\partial y}dy$$

## 3 Entropy analysis

The entropy serves as a thermodynamic potential, providing a measurable indication of irreversibility. The mathematical description of the volumetric entropy generation rate is

(33) $$S_{G}=\frac{\bar{K}\left(T\right)}{{\left(T_{1}-T_{o}\right)}^{2}}\left\{\bar{\nabla }T.\bar{\nabla }T\right\}+\frac{1}{\left(T_{1}-T_{0}\right)}\left[\bar{s}.L\right]+\frac{1}{\left(T_{1}-T_{0}\right)}\left[\sigma_{f}{\bar{U}}^{2}B_{0}^{2}\right]+\frac{R^{*}D}{\left(T_{1}-T_{0}\right)}\left(\frac{\partial C}{\partial \bar{X}}\frac{\partial T}{\partial \bar{X}}+\frac{\partial C}{\partial \bar{X}}\frac{\partial T}{\partial \bar{X}}\right)+\frac{R^{*}D}{\left(C_{1}-C_{0}\right)}\left({\left(\frac{\partial C}{\partial \bar{X}}\right)}^{2}+{\left(\frac{\partial C}{\partial \bar{Y}}\right)}^{2}\right)\text{.}$$

Here the initial term explains the irreversible nature of heat transfer in the context of entropy production, the subsequent term pertains to fluid friction, the third term signifies the magnetic field, and the final term represents concentration, $S_{G}$ is contributing to the local volumetric entropy generation rate.

The current model's entropy generation number is provided by

(34) $$N_{s}=\frac{S_{G}}{S_{c}}$$

where

(35) $$S_{c}=\frac{K_{o}}{d_{1}^{2}}$$

in which $S_{C}$ is the characteristic rate of entropy generation.

With the help of Eqs. (33), (35) in Eq. (36), the entropy generation rate in the dimensionless form is:

(36) $$N_{s}={\left(\frac{\partial \theta }{\partial y}\right)}^{2}\left(1+\xi \theta \right)+Br\varphi +{\left(\frac{\partial \psi }{\partial y}\right)}^{2}BrM^{2}+L\frac{\partial \theta }{\partial y}\frac{\partial \varphi }{\partial y}+L\frac{\partial \theta }{\partial y}\frac{\partial \varphi }{\partial y}+L{\left(\frac{\partial \varphi }{\partial y}\right)}^{2}$$

in which $L=\frac{R^{*}D\left(C_{1}-C_{0}\right)}{k_{o}}$ designates the diffusion parameter.

The Bejan number (Be) is the entropy production ratio which defines the ration of heat transportation irreversible to the total entropy production as

(37) $$Be=\frac{{\left(\frac{\partial \theta }{\partial y}\right)}^{2}\left(1+\xi \theta \right)}{{\left(\frac{\partial \theta }{\partial y}\right)}^{2}\left(1+\xi \theta \right)+Br\varphi +{\left(\frac{\partial \psi }{\partial y}\right)}^{2}BrM^{2}+L\frac{\partial \theta }{\partial y}\frac{\partial \varphi }{\partial y}+L\frac{\partial \theta }{\partial y}\frac{\partial \varphi }{\partial y}+L{\left(\frac{\partial \varphi }{\partial y}\right)}^{2}}$$

## 4 Numerical method

The MATLAB bvp4c numerical method is employed to solve a non-linear system of Ordinary Differential Eqs. (ODEs) (refer to Eqs. (22)–(26)), incorporating boundary constraints outlined in Eq. (28) and (29). These transformed equations are represented as higher-order ODEs, which are then converted into a first-order form initially is

$$f=y_{1},f^{\prime }=y_{2}\text{,}$$

$$g=y_{3},g^{\prime }=y_{4}\text{,}$$

$$\theta =y_{5},\theta^{\prime }=y_{6}\text{,}$$

$$\varphi =y_{7},\varphi^{\prime }=y_{8}\text{,}$$

$$y_{4}^{\prime }=\frac{1}{1+2c_{1}y_{3}}\left(-\left(1+2c_{1}\right)y_{4}^{2}+M^{2}y_{3}\right)-Gry_{6}-2Gc\left(1+c_{1}\right)y_{3}^{2}y_{4}-c_{1}y_{3}^{2}y_{4}+k^{3}U_{hs}\frac{\cosh\left(ky\right)}{\sinh\left(2kh\right)}$$

$$y_{6}^{\prime }=\frac{1}{-1-\varsigma y_{5}}\left(\zeta y_{6}^{2}+BrM^{2}y_{2}^{2}+Nb\;\mathrm{Pr}\;y_{8}y_{6}+NrBry_{6}^{2}+Bry_{5}\right)$$

$$y_{8}^{\prime }=\frac{Nt}{Nb}y_{6}^{\prime }$$

The transformed boundary conditions are

$$y_{1}\left(0\right)+\frac{F}{2}=0,y_{2}\left(0\right),y_{5}\left(0\right)-\frac{1}{2},y_{7}\left(0\right)-\frac{1}{2}$$

$$y_{1}\left(\infty \right)-\frac{F}{2}=0,y_{2}\left(\infty \right),y_{5}\left(\infty \right)+\frac{1}{2},y_{7}\left(\infty \right)+\frac{1}{2}$$

### 4.1 Validation of computational results

The axial velocity values calculated using the MATLAB bvp4c command were cross-referenced with the data from a study by F.M. Abbasi et al. [35]. Upon examination of Table-1, it is evident that the axial velocity results obtained in the current research closely align with those of [35] under conditions without osmosis.

**Table 1:** Comparison between existing literature [35] and numerical solution of the present study for different value of *y.*

*y*	Axial velocity (*u*) for existing literature [35]	Axial velocity (*u*) for present numerical solution	Absolute Error
0	−0.989173	−0.988479	0.00069
0.2	−0.989167	−0.988479	0.00062
0.4	−0.992762	−0.992291	0.00047
0.6	−0.992770	−0.992299	0.00047
0.8	−0.992784	−0.992312	0.00047
1	−0.992801	−0.992330	0.00047

## 5 Graphical description

In this section, we will present the computational results for the axial velocity function, temperature function, nanoparticles fraction function, entropy generation function, and Bejan number through an asymmetric micro channel, explain and analyse them, and provide a physical interpretation of the results. MATLAB bvp4c commands are used to compute numerical results. The following fixed values adopted to draw the plots.

$$\begin{array}{c} n=2.0,t=0.2,We=0.3,x=0.4,a=0.4,b=0.3,Nt=0.5,\mathrm{Pr}=0.7\text{,}\\ m=0.2,Nb=0.5,Gr=2.0,Gc=2.0,\varepsilon =\frac{\pi }{4},\beta =0.2,\eta =0.2,Br=0.3\text{,}\\ M=2,F=2,Nr=0.5,k=1,Uhs=0.2,L=0.2 \end{array}$$

In the various subsections of this graphical discussion section that follow, we visualize and simulate the variations in the flow fields, such as dimensionless fluid temperature θ(ζ), fluid velocity $f^{\prime }\left(\zeta \right)$, entropy generation Ns, nanoparticles fraction function φ(ζ), and the Bejan number Be versus major governing flow variables.

### 5.1 Velocity function $f^{\prime }$(ζ)

**Fig. 2:**
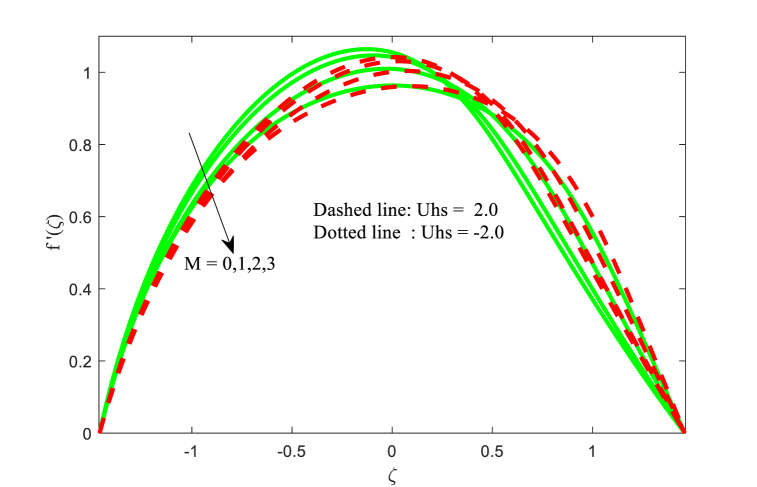
Variation in u with M.

**Fig. 3:**
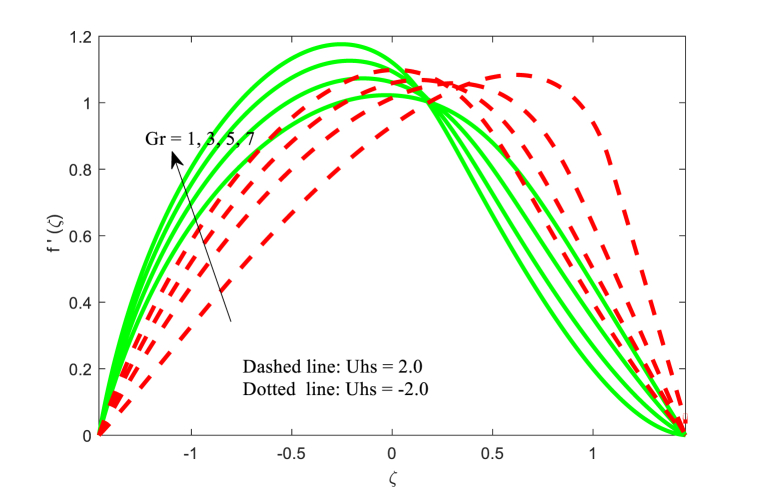
Variation in u with Gr.

**Fig. 4:**
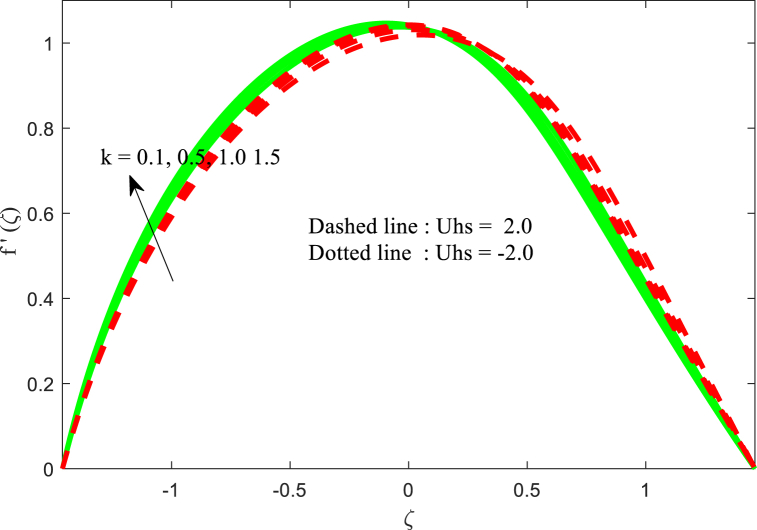
Variation in u with k.

Fig. 2 depicts the effect of the magnetic field effect on the velocity distribution function $f^{\prime }\left(\zeta \right)$ for two cases of Smoluchowski - Helmholtz velocity Uhs. The field of electrical in the fluid movement direction is indicated in the negative values of Uhs, and the field of electrical in the opposite direction of the non-uniform channel is indicated by the positive values of Uhs. The dimensionless velocity is diminishing near the center of channel for boosting values of M whereas the opposite trend (i.e., reverse trend) is found near the right wall of the channel. This physical situation agrees due to magnetic field enhances the Lorentz force (resistance force) and results opposes the fluid flow in the channel. This physical situation exists because the magnetic field enhances the resistance force (Lorentz force), which opposes the fluid flow in the channel. Fluid velocity is found to be greatest in the channel's centre at negative values of Uhs. It is noted that the same physical situation is found in Refs. [49,50]. Furthermore, the fluid velocity reduced for larger Helmholtz-Smoluchowski velocity parameter Uhs. Fig. 3 is presented to examine the interaction of thermal Grashof number Gr on the fluid velocity field. It is revealed that the fluid velocity increases for higher Grashof number Gr for both negative and positive values of Uhs. The influence of electro osmotic parameter k (Debye-Hückel parameter) on the non-dimensional velocity for both positive and negative values of Uhs has been reported in Fig. 4. It is evident that the velocity behaves differently near the channel walls compared to where it is located in the middle of the non-uniform channel. It is also worth noting that fluid velocity increases and decreases on a regular basis, raising the values of the Debye-Hückel parameter k in both cases of Helmholtz-Smoluchowski velocity Uhs. A comparable pattern can be found in the existing literature [35].

### 5.2 Temperature function θ(ζ)

**Fig. 5:**
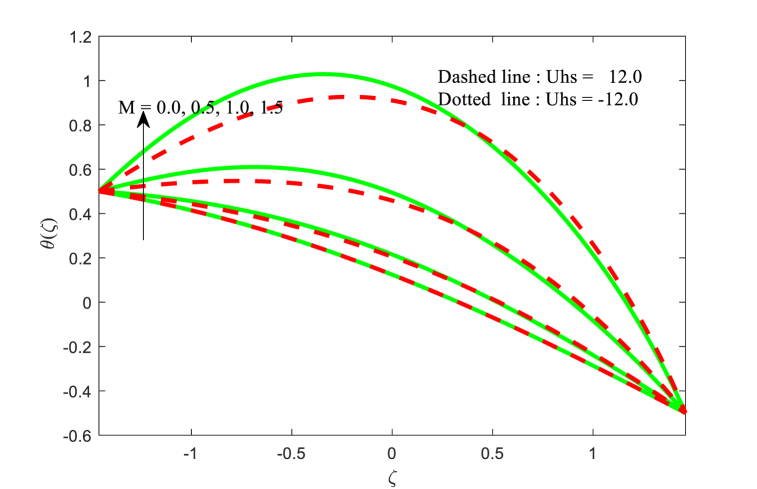
Variation in θ with M.

**Fig. 6:**
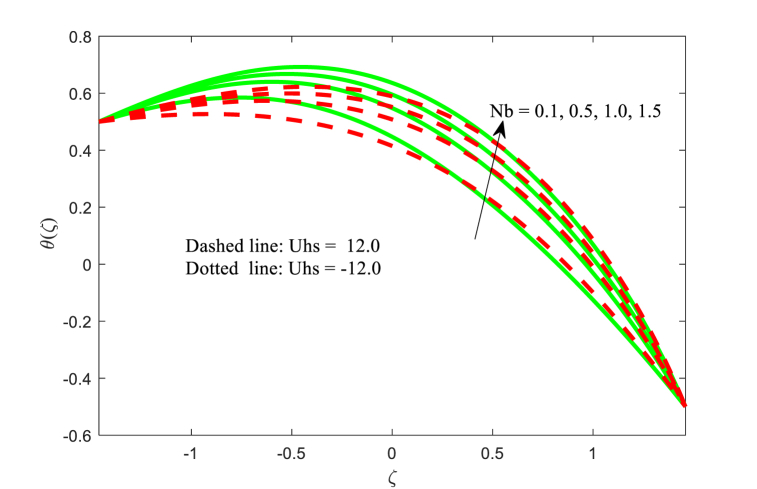
Variation in θ with Nb.

**Fig. 7:**
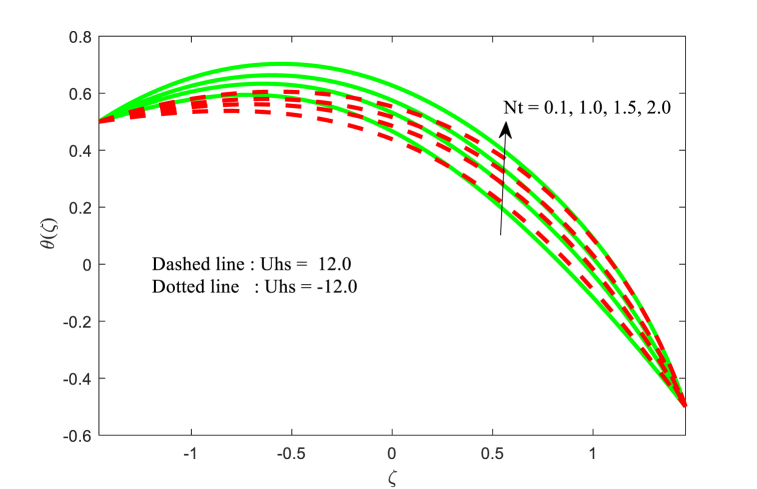
Variation in θ with Nt.

**Fig. 8:**
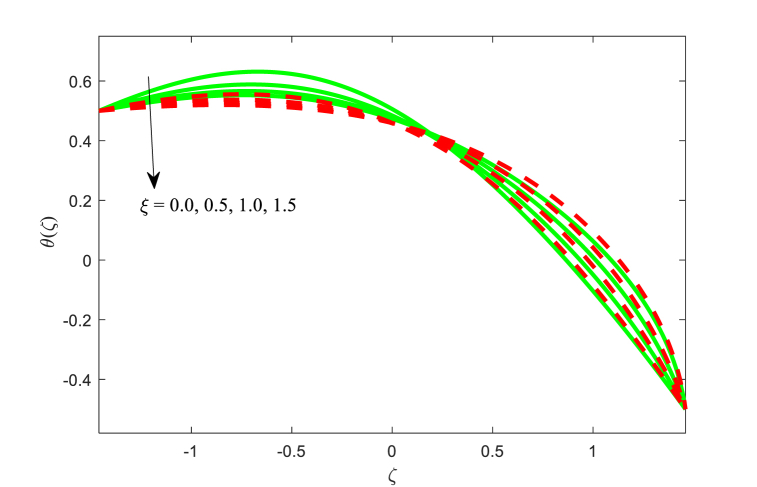
Variation in θ with ξ.

The temperature function θ(ζ) against ζ for the effect of Brownian motion parameter Nb, magnetic field parameter M, thermophoresis parameter Nt, and thermal conductivity parameter ξ with two variations of Helmholtz-Smoluchowski velocity Uhs are displayed in Fig. 5, Fig. 6, Fig. 7, Fig. 8 correspondingly. From Fig. 5, it is noticed that the fluid temperature is boosting for higher applied magnetic field in the non-uniform channel. In addition, the temperature transport of the fluid is maximum at central part of the channel for positive value of Uhs. Fig. 6 makes it clear that when the Brownian motion increases, the fluid temperature increases throughout the channel. From Fig. 7, it is inspected that temperature of the fluid is increasing with increase in thermophoresis. Also, the impact of Helmholtz-Smoluchowski velocity for the thermophoresis is more as compared to the Brownian motion (see Fig. 6, Fig. 7). Furthermore, the influence of thermophoresis and Brownian motion for the temperature function is having similar trend at the walls of the channel. The effect of conductivity of thermal parameter ξ on the non-dimensional temperature function is observed in Fig. 8. It can be studied that the temperature is reduced for larger thermal conductivity parameter ξ near the left wall while the contrast effect for another wall of the channel.

### 5.3 Nanoparticles fraction function

**Fig. 9:**
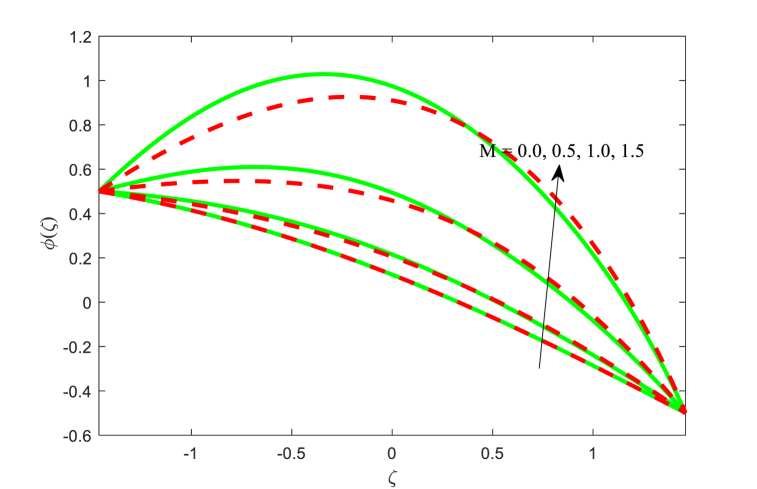
Variation in φ with M.

**Fig. 10:**
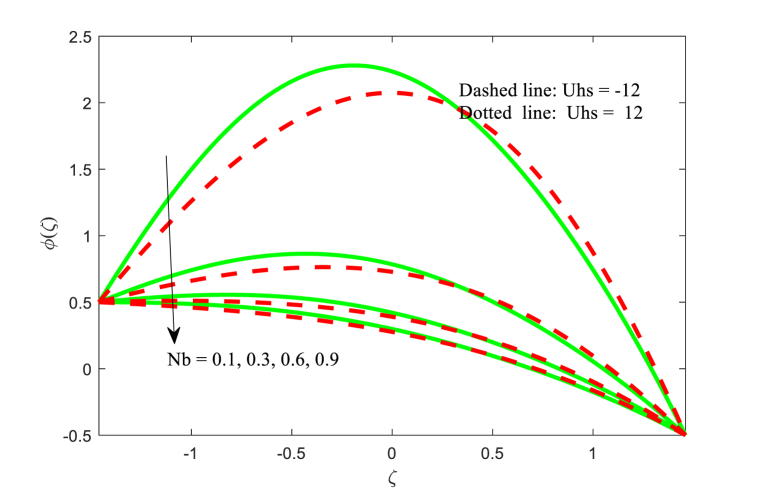
Variation in φ with Nb.

**Fig. 11:**
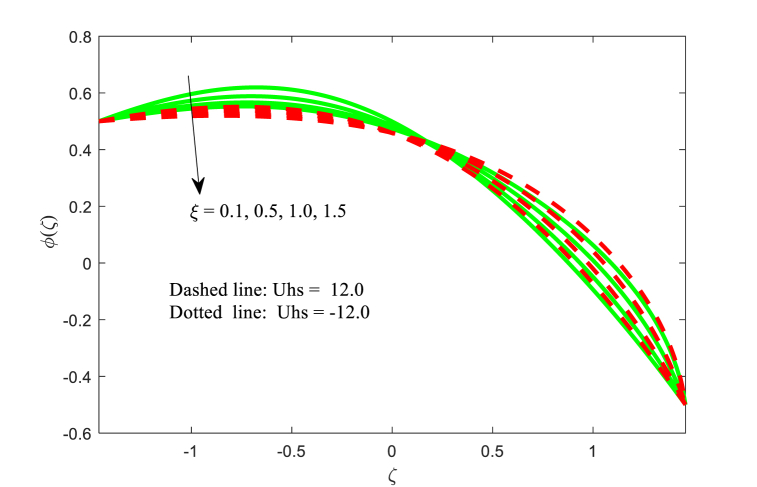
Variation in φ with ξ.

The effect of physical flow variables such as Hartmann number M, Brownian motion parameter Nb and variable thermal conductivity parameter ξ on the nanoparticle fraction function φ(ζ) have been scrutinized in Fig. 9, Fig. 10, Fig. 11. It is seen from Fig. 9 that as higher magnetic field, nanoparticle fraction function φ(ζ) is increased throughout the channel. Also, the impact of Hartmann number on both fluid temperature and nanoparticle fraction φ(ζ) is same trend while the opposite effect is inferred for the dimensionless velocity function. It is revealed from Fig. 10 that the nanoparticle fraction profile φ(ζ) is diminishing function for the Brownian motion parameter Nb. For smaller values of Brownian motion, the nanoparticle fraction is high as compared to boosting of. The nanoparticle fraction distribution for the effect of thermal conductivity parameter ξ is plotted in Fig. 11. The nanoparticle fraction field reduced for escalating the variable thermal conductivity parameter ξ near the left wall of the non-uniform channel. Also, the nanoparticle fraction function is lower for the absence of variable thermal conductivity parameter (i.e., ξ=0) at near the right wall of the channel.

### 5.4 Entropy generation *Ns*

**Fig. 12:**
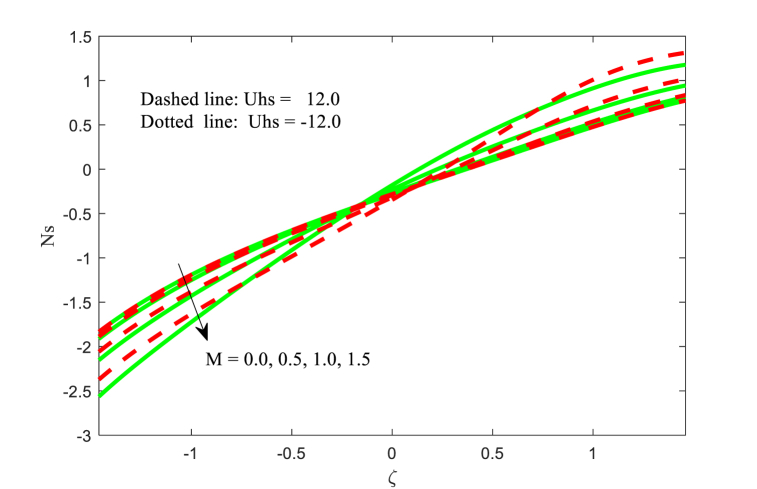
Variation in Ns with M.

**Fig. 13:**
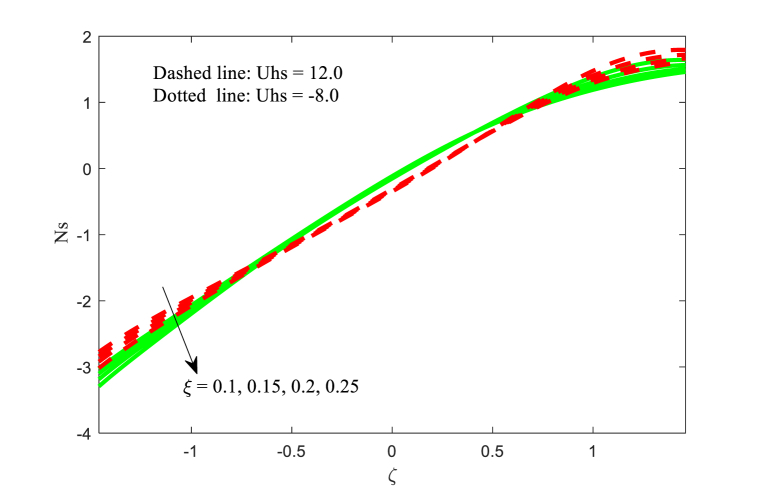
Variation in Ns with ξ.

The entropy generation Ns for the effect of Hartmann number M and variable thermal conductivity parameter ξ with two variations of Helmholtz-Smoluchowski velocity Uhs are presented in Fig. 12, Fig. 13. The entropy generation are boosts for the Hartmann number M near the right wall of the channel while the opposite trend is observed at the other wall of the channel from Fig. 12. It is noticed that fluid entropy generation is minimum at left wall of the channel for negative values of Uhs. The similar physical behaviour is identified for the variable thermal conductivity parameter ξ (see Fig. 13). In addition, the entropy generation function is more at the central part of the channel for negative values of Uhs. The concept of energy balance, as per thermodynamic principles, posits that energy cannot be generated or eliminated but may be conserved or transformed into different forms like heat. In a similar vein, in cyclic loading scenarios, a significant portion of energy is expended and extracted from the system as the material undergoes plastic deformation, leading to irreversible changes in its microstructure. These irreversible processes contribute to entropy generation, a factor that plays a role in fatigue crack formation, being a non-reversible process.

### 5.5 Bejan number **Be**

**Fig. 14:**
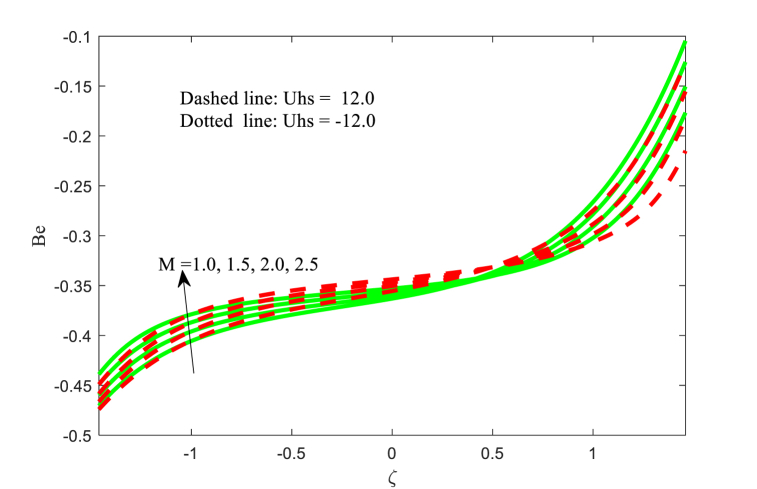
Variation in Be with M.

**Fig. 15:**
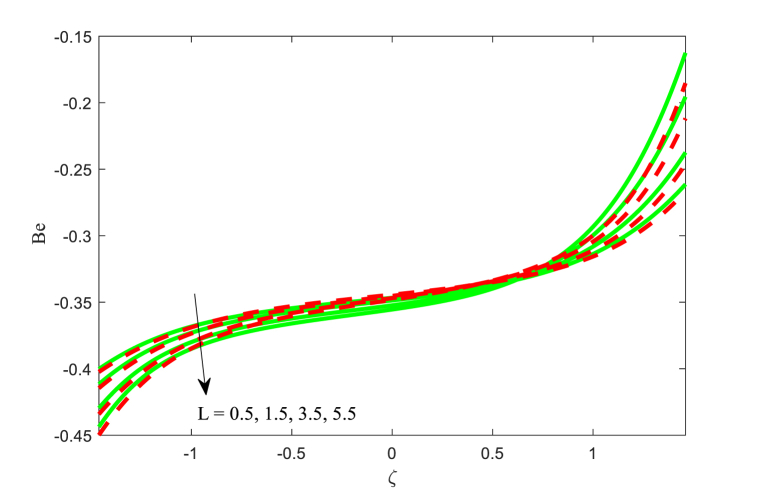
Variation in Be with L.

**Fig. 16:**
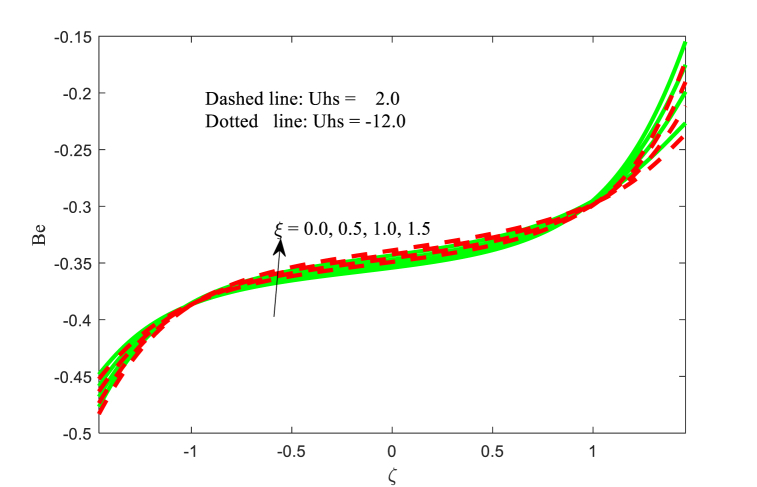
Variation in Be with ξ.

Fig. 14, Fig. 15, Fig. 16 scrutinize the effect of Hartmann number M, slip parameter L, variable thermal conductivity parameter ξ on the Bejan number Be are elaborated in this section. It is inferred from Fig. 14 that the Bejan number Be is increasing behaviour for higher applied magnetic field strength near the left wall of the channel. The impact of M at the right wall of the channel is more considerable variation as contrasted to the other wall. The similar physical behaviour is observed in Fig. 15. The influence of variable thermal conductivity parameter ξ on the Bejan number Be at the both walls and centre of the channel are different and is evident from Fig. 16. Finally, the Bejan number function is favored at the right wall of the channel while the reverse trend is found at another wall of the non-uniform channel. The change in entropy production is minimal close to the core but more significant near the channel wall. The reason for this phenomenon lies in the limited transfer of energy near the walls by the fluid movement instead of the central region, resulting from elevated temperatures close to the wall surface. The reverse relationship between Bejan Number and entropy generation detected.

### 5.6 Streamlines discussion

**Fig. 17:**
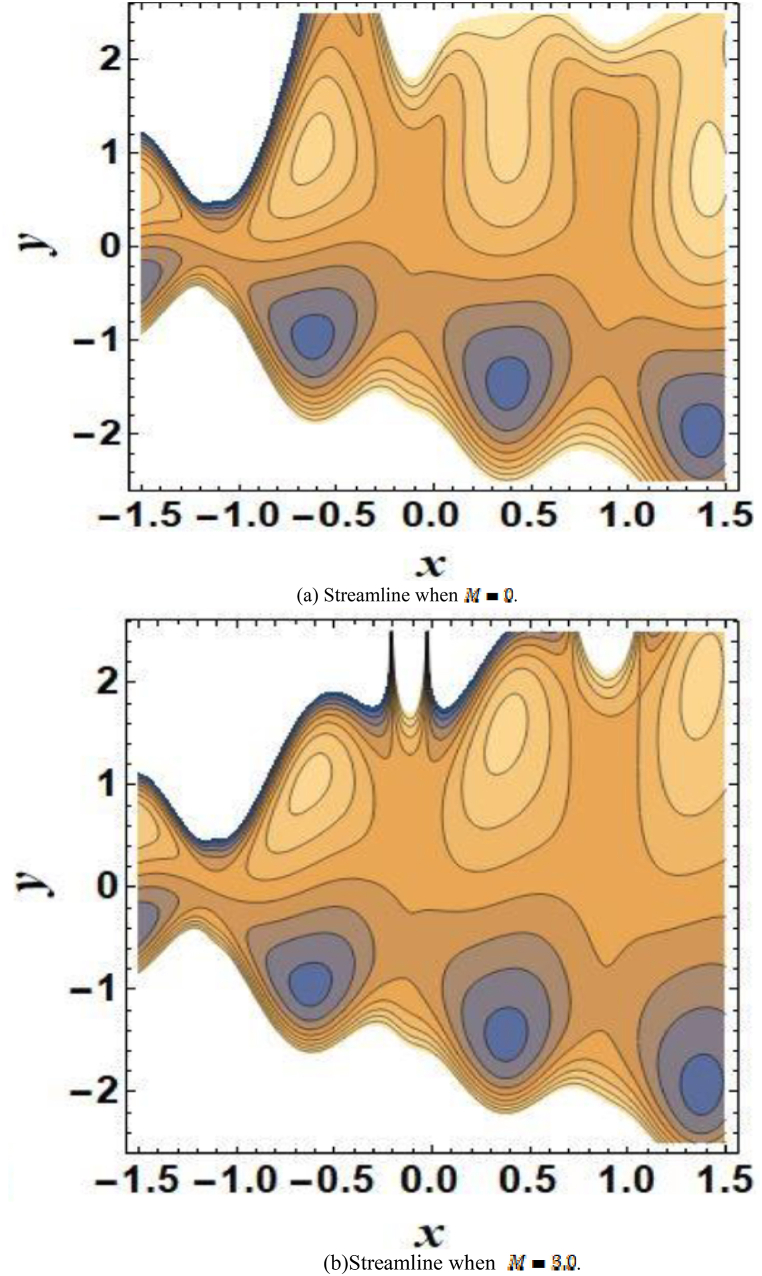
(a) Streamline when M=0.Fig. 17. (b)Streamline when M=3.0.

Fig. 17 illustrates the formation of circulating bolus inside the fluid mass as an outcome of the trapping phenomenon. The peristaltic wave is drawn forward with the trapped bolus. The streamlines for Hartmann number (M) is presented in Fig. 17(a–b). From Fig. 17(a–b), it is noticed that as the Hartmann number increases, the size of internal bolus increases. The cohesive forces among fluid particles within the uneven microchannel diminish as a result of fluid movement caused by the reversed distribution of electrical charges. This process is reversed when the flow direction aligns with the movement of electrical charges.

## 6 Conclusions

The interactions of entropy generation on hydromagnetic Nanofluid flow induced by peristaltic wave through a non-uniform asymmetric channel via electro-osmotic were examined. The present work analyzes entropy generation with nanofluid and further develops entropy generation with hybrid nanofluid and ternary nanofluid. The computations have shown that:

1. The behaviour of Brownian motion and thermophoresis on the temperature distribution exhibits a consistent pattern near the channel walls.
2. The non-dimensional speed decreases close to the channel's midpoint for elevated values of M. Conversely, a different pattern is observed near the channel's right-side boundary.
3. Entropy function boosts for the Hartmann number M near the right wall of the channel while the opposite trend is observed at the other wall of the channel.
4. Bejan number function is boosted at the right wall of the channel whereas the reverse trend has been found at another wall of the non-uniform channel.
5. The entropy generation is minimum at left wall of the channel for negative values of Helmholtz-Smoluchowski velocity.
6. The nanoparticle fraction field reduced for escalating the variable thermal conductivity parameter ξ near the left wall of channel.

## Funding statement

No financial assistance for the research described in the article.

## Data availability statement

No data was used for the research described in the article.

## CRediT authorship contribution statement

**M. Gnaneswara Reddy:** Software. **K. Venugopal Reddy:** Methodology. **Basma Souayeh:** Data curation. **H. Fayaz:** Writing – review & editing.

## Declaration of competing interest

The authors declare that they have no known competing financial interests or personal relationships that could have appeared to influence the work reported in this paper.

## Nomenclature

**Symbol Description**
$\left(a_{1},b_{1}\right)$: amplitude of the asymmetric micro channel's both sides (m)
e: fundamental charge
(a,b): the asymmetric micro channel's left and right non-dimensional amplitude
d: channel of width (m)
$\bar{E}$: potential of electrical
$c_{p}$: heat specific capacity (J/(kg. K)) (Kelvin^−1^ s^−2^ m^2^)
g: acceleration due to gravity (*ms*^−1^)
$\left(C_{0},C_{1}\right)$: Concentration at the left and right walls respectively
$\bar{C}$: Concentration
E: dimensionless electric potential
c: speed of wave (m/s)
$D_{B}$: The coefficient of Brownian diffusion coefficient (m^2^ s^−1^)
$E_{x}$: applied electrical field
$G_{t}$: Grashof thermal number
$G_{c}$: Grashof number
M: Hartman number
$\left(T_{1},T_{0}\right)$: Temperature at the right and left walls respectively (*Kelvin*)
$U_{HS}$: Helmholtz-Smoluchowski velocity
We: Weissenberg number
$D_{T}$: The coefficient of thermophoretic diffusion (*m*^2^*sec*^−1^)
Pr: Prandtl number
Ec: Eckert number
$\left(\bar{u},\bar{v}\right)$: velocity fixed frame components of $\left(x^{*},y^{*}\right)$
(u,v): Non-dimensional components of velocity (x,y)
$S_{G}$: The rate of volumetric local entropy generation
$S_{C}$: The rate of entropy characteristic generation
$\mu_{o}$: zero shear rates of viscosity
φ: phase difference
$\mu_{\infty }$: infinite shear rates of viscosity
Be: Bejan number

**Greek symbols**
$\rho_{p}$: nanoparticle density of mass (kg/m^3^)
${\bar{\rho }}_{e}$: electric charge density per unit volume
μ: The viscosity of fluid dynamics (kg m^−1^ s^−1^)
λ: wavelength (m)
$\sigma^{*}$: Stefan-Boltzmann constant (W m^−2^ Kelvin^−4^)
$\rho_{o}$: The density of nanofluid at the reference temperature (kg/m^3^)
ε: dielectric permittivity of the medium (Fm^−1^)
$\rho_{f}$: nanoparticle fluid density (kg/m^3^)
κ: electro-osmotic parameter (Debye length)
ψ: stream function
ς: zeta potential (v)
$\xi_{o}$: dimensional thermal conductance
